# Blood-Flow-Restriction-Training-Induced Hormonal Response is not Associated with Gains in Muscle Size and Strength

**DOI:** 10.2478/hukin-2022-0095

**Published:** 2022-09-08

**Authors:** Gilberto C. Laurentino, Jeremy P. Loenneke, Carlos Ugrinowitsch, Marcelo S. Aoki, Antonio G. Soares, Hamilton Roschel, Valmor Tricoli

**Affiliations:** 1 School of Physical Education and Sport, University of São Paulo, SP, Brazil; 2 Department of Health, Exercise Science, and Recreation Management, Kevser Ermin Applied Physiology Laboratory, The University of Mississippi, MS, USA; 3 School of Arts, Sciences, and Humanities, University of São Paulo, SP, Brazil; 4 Institute of Biomedical Science, University of São Paulo, SP, Brazil

**Keywords:** resistance training, anabolic hormones, hypertrophy, vascular occlusion, cross-sectional area, muscle force

## Abstract

The aim of this study was to determine whether increases in post-exercise endocrine response to low-load resistance exercise with blood flow restriction and high-load resistance exercise would have association with increases in muscle size and strength after an 8-week training period. Twenty-nine untrained men were randomly allocated into three groups: low-load resistance exercise with (LL-BFR) or without blood flow restriction (LL), and high-load resistance exercise (HL). Participants from LL-BFR and LL groups performed leg extension exercise at 20% of one repetition maximum (1RM), four sets of 15 repetitions and the HL group performed four sets of eight repetitions at 80% 1RM. Before the first training session, growth hormone (GH), insulin-like growth factor 1 (IGF-1), testosterone, cortisol, and lactate concentration were measured at rest and 15 min after the exercise. Quadriceps CSA and 1RM knee extension were assessed at baseline and after an 8-week training period. GH increased 15 min after exercise in the LL-BFR (p = 0.032) and HL (p < 0.001) groups, with GH concentration in the HL group being higher than in the LL group (p = 0.010). There was a time effect for a decrease in testosterone (p = 0.042) and an increase in cortisol (p = 0.005), while IGF-1 remained unchanged (p = 0.346). Both muscle size and strength were increased after training in LL-BFR and HL groups, however, these changes were not associated with the acute post-exercise hormone levels (p > 0.05). Our data suggest that other mechanisms than the acute post-exercise increase in systemic hormones induced by LL-BFR and HL produce changes in muscle size and strength.

## Introduction

Low-load resistance exercise (20 to 30%) of one repetition maximum (1RM) in combination with blood flow restriction (LL-BFR) has been demonstrated to induce increases in muscle size and strength to a similar level to that observed with high-load resistance exercise (≥65-70% 1RM) (Ellefsen et al., 2015; Laurentino et al., 2012; Takarada et al., 2000).

Localized hypoxia seems to be the primary candidate mechanism responsible by the anabolic response to LL-BFR (Fry et al., 2010; Fujita et al., 2007). In this regard, metabolite accumulation, leading to greater muscle fiber recruitment has been widely reported in the literature (Gonzales et al., 2016; Rawska et al., 2019; Reeves et al., 2005; Takarada et al., 2000). Similarly, anabolic (Fry et al., 2010; Fujita et al., 2007) and anti-catabolic signaling (Laurentino et al., 2012), as well as activation and proliferation of myogenic stem cells (Nielsen et al., 2012) have been shown as results of this type of training. Finally, a significant amount of attention has been devoted to the endocrine response to BFR. Some studies have demonstrated elevated levels of growth hormone (GH) caused by an accumulation of metabolites (e.g., lactate), and insulin-like growth factor 1 (IGF-1) after LL-BFR, which are thought to mediate the anabolic responses to training (Abe et al., 2005; Manini et al., 2012; Takano et al., 2005). For instance, a recent study showed increases in serum GH, testosterone, and IGF-1 response after a LL-BFR protocol at 30% 1RM associated to 40% and 70% of arterial occlusion pressure, with concomitant increased lactate concentration (Yingao et al., 2021).

Importantly, although the alleged anabolic role of acute endocrine responses to conventional resistance training has been strongly challenged (West et al., 2009, 2010; West and Phillips, 2012; Wilkinson et al., 2006), little is known regarding the possible association between acute endocrine responses to LL-BFR and chronic-induced increases in muscle size and strength. Therefore, the purpose of this study was to investigate whether there is a relationship between the acute hormonal response and accumulation of metabolites (e.g., lactate) observed after a single session of LL-BFR with morphological and functional adaptations to an 8-week LL-BFR training program. The secondary purpose was to investigate whether these hormonal responses are comparable to those observed with both low-load resistance exercise (LL) and conventional high-load resistance exercise (HL). Although we expected acute elevation in hormone levels and lactate concentration, we hypothesized that there would be no association between them and the increases in muscle size and strength.

## Methods

### Participants

Twenty-nine physically active male college students were randomly allocated into three groups: low-load resistance exercise with blood flow restriction (LL-BFR, n = 10, age = 20.0 ± 4.5 yr, body mass = 72.1 ± 11.9 kg, body height = 175.2 ± 9.0 cm), low-load resistance exercise (LL, n = 10, age = 20.3 ± 4.2 yr, body mass = 75.3 ± 15.4 kg, body height = 175.7 ± 4.9 cm), and high-load resistance exercise (HL, n = 9, age = 23.6 ± 6 yr, body mass = 73.8 ± 12 kg, body height = 173.6 ± 6 cm). Participants were not engaged in any form of regular physical training and had no previous experience in strength training. They were free from any musculoskeletal disorders and drug or nutritional supplements ingestion. Participants were informed of the benefits and potential risks of the investigation and gave their written informed consent prior to the commencement of the study. The study was conducted according to the Declaration of Helsinki and the University's Research Ethics Committee approved the experimental protocol.

### Experimental Design

After two familiarization sessions with a bilateral knee extension exercise, a one repetition maximal test (1RM) and blood flow restriction protocols, participants from LL, LL-BFR, and HL groups performed their respective training protocols. Blood samples were taken before the first session of exercise (rest) and 15 min after completing the exercise session (15 min) for determination of GH, IGF-1, testosterone, cortisol, and lactate concentrations. The quadriceps cross-sectional area (CSA) and knee extension 1RM were assessed before and after 8 weeks of training by magnetic resonance imaging technique. Pearson´s correlation coefficients were calculated to determine the relationship between the acute absolute changes of hormones and lactate concentrations and CSA and 1RM measurements.

### Blood Samples for Hormones

Prior to testing, participants rested in a supine position for at least 30 min, and then, blood samples were collected at 8:00 - 10:00 a.m. using an indwelling heparin lock catheter which was inserted into the superficial antecubital vein of the left arm for the determination of GH, IGF-1, testosterone, and cortisol concentrations. Blood samples were taken before starting the exercise (rest) and 15 min after the exercise session (15 min). For hormone measurements, blood samples (5 ml) were drawn into test tubes containing EDTA. All blood samples were kept in ice-cold water and centrifuged (10000 rpm) for 10 min and the plasma was removed and stored frozen at - 80°C until the assays were performed. Plasma hormone concentrations were determined by a commercially available enzyme-linked immunosorbent assay (ELISA). Data of GH of five participants from three groups were excluded from the analysis due to technical error: two from the LL group, one from the LL-BFR group and two from the HL group. In addition, two participants of the HL group were excluded from IGF-1 analysis.

### Lactate Concentration

Measurements of blood lactate concentration were analysed for each experimental protocol at rest and 15 min after exercise. After local asepsis with alcohol, a small earlobe incision was done using a disposable lancet to collect the blood. An arterial blood sample (25μl) was collected and inserted in a capillary tube. Blood lactate concentrations were measured by an electro-enzymatic lactate analyzer (1500 Sport, Yellow Springs Instruments, Yellow Springs, USA), which was previously calibrated with a known concentration of lactate of 5 mmol∙L-1.

### Quadriceps Cross-Sectional Area (CSA)

The quadriceps CSA was obtained through magnetic resonance imaging (MRI - Signa LX 9.1; GE Healthcare, Milwaukee, WI) and measured at 50% of the femur length. The images were then transferred to a workstation (Advantage Workstation 4.3; GE Healthcare) and were traced by a specialized researcher. Mean values from three measures were used for further analysis.

### Knee Extension 1RM Test

For knee extension 1RM determination, after participants ran for 5 min on a treadmill at 9 km∙h^-1^, one set of eight repetitions at approximately 50% 1RM was performed, followed by the second set of three repetitions at approximately 70% 1RM with a 2 min rest interval between trials. After that, participants were allowed up to five attempts to achieve their 1RM with a 3-min rest interval between trials. The greatest 1RM weight lifted was considered for further analysis.

### Arterial Occlusion Pressure Determination

Participants were asked to lie in a supine position while resting comfortably. A vascular handheld Doppler probe was placed over the tibial artery to capture its auscultatory pulse. For the determination of blood pressure (mmHg) necessary for a complete vascular restriction (pulse elimination pressure), a standard blood pressure cuff was attached to the participant’s thigh (inguinal fold region) and then inflated up to the point in which the auscultatory pulse was interrupted (Laurentino et al., 2012). The cuff pressure used during the training protocol was determined as 80% of the necessary pressure for the complete blood flow restriction in a resting condition. The average pressure used throughout the training protocol was 94.8 ± 10.3 mm Hg. The arterial occlusion pressure intraclass correlation coefficient (ICC) and the coefficient of variation (CV) were 0.938 and 1.1%, respectively.

### Exercise Protocols

After a general warm-up on a treadmill at 9 km∙h^-1^ for five minutes, participants from LL, LL-BFR, and HL groups performed bilateral knee extension protocols. Participants from LL and LL-BFR groups performed four sets of 15 repetitions at 20% 1RM. For the LL-BFR protocol a nylon cuff (17.5 cm width x 92 cm length) was placed at the most proximal region of the thigh and inflated at 80% of arterial occlusion pressure and removed immediately after the protocol. Participants from the HL group performed four sets of eight to ten repetitions at 80% 1RM. The LL and LL-BFR protocols were performed with a 60 s rest interval between sets, while the HL protocol was performed with a 90 s rest interval between sets. All protocols were performed with a tempo of 1 s for the concentric and 1 s for the eccentric phases. Participants performed training sessions twice a week, for 8 weeks, with supervision of an experienced researcher. Only one participant withdrew from the HL group for personal reasons.

### Statistical Analysis

Data are presented according to descriptive statistics as means and standard deviations. The Shapiro-Wilk test was used to assure that the data was normally distributed. A mixed model for repeated measures was applied, using as fixed factors the experimental groups (LL, LL-BFR, and HL) and times (pre- and 15 min post-exercise) for acute hormonal responses (GH, IGF-1, testosterone, and cortisol) and lactate concentrations. When a significant F value was obtained, the Tukey post hoc test was used for multiple comparisons. Pearson´s correlation coefficients were calculated to determine the relationship between the acute absolute changes (pre- and 15 min post-exercise) in the measured variables [GH (ΔGH), IGF-1 (ΔIGF-1), testosterone (Δtestosterone), cortisol (Δcortisol), and Δlactate] with chronic absolute changes (pre- and post-8-week training) in the CSA (ΔCSA) and 1RM (Δ1RM). The level of significance was set at *p* ≤ 0.05, and all analyses were conducted with the statistical software package SAS 9.2. (SAS Institute Inc., Cary, NC, USA).

## Results

**Figure 1:**
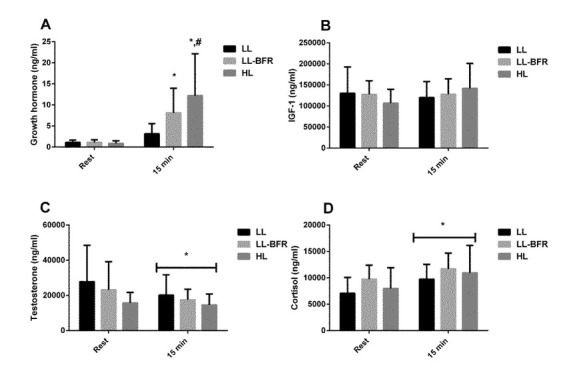
Acute changes in growth hormone [GH] (A), insulin-like growth factor 1 [IGF-1] (B), testosterone (C) and cortisol (D) concentrations from rest to 15 min after exercise, in the low-load resistance exercise (LL), low-load resistance exercise with blood flow restriction (LL-BFR), and high-load resistance exercise (HL) groups. (*) indicates p < 0.05 from rest. (#) indicates p < 0.05 compared to the LL group.

There was a significant time x condition interaction for GH (*p* = 0.037). GH concentration was increased 15 min after exercise in the LL-BFR (*p* = 0.032) and HL (*p* = 0.0009) groups (Figure 1A). In addition, GH concentration 15 min after exercise was higher in the HL group than in the LL group (*p* = 0.010). There were no significant condition x time interactions for testosterone and cortisol, but there was a main effect of time for testosterone (*p* = 0.042) and cortisol (*p* = 0.005) (Figures 1C and 1D). There were no significant changes in IGF-1 concentration (Figure 1B).

**Figure 2:**
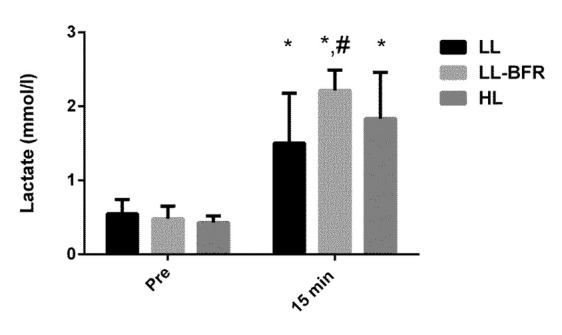
Acute changes in lactate concentration from rest to 15 min after exercise in the low-load resistance exercise (LL), low-load resistance exercise with blood flow restriction (LL-BFR), and high-load resistance exercise (HL) groups. (*) indicates p < 0.05 from rest. (#) indicates p < 0.05 compared to the LL group.

There was a significant time x condition interaction for lactate concentration (*p* = 0.016). Lactate concentrations were increased 15 min after exercise in the LL, LL-BFR, and HL groups (*p* = 0.0001); however, lactate concentration was significantly higher in the LL-BFR than in the LL group 15 min after exercise (*p* = 0.008) (Figure 2).

As expected, 8 weeks of training resulted in significant gains in the muscle CSA (LL pre: 8188 ± 1358, post: 8265 ± 1173, *p* = 0.965; LL-BFR pre: 7720 ± 1298, post: 8182 ± 1230, *p* < .0001; HL pre: 7572 ± 1832, post: 8030 ± 1983 mm^2^, *p* < .0001) and 1RM (LL pre: 86.2 ± 9.5, post: 106.7 ± 10.8, *p* < .0001; LL-BFR pre: 84.7 ± 14.5, post: 118.7 ± 16.4, *p* < .0001; HL pre: 86.9 ± 15.2, post: 118.3 ± 18.9 kg, *p* < .0001). Moreover, increases in muscle mass (~6.3% vs. 6.1%) and strength (40% vs. 36%) were similar in the LL-BFR and HL groups, respectively, whereas LL resulted in much smaller strength increments (~23%) with no changes in the CSA. However, there was no significant difference between groups in the muscle CSA and 1RM (*p* > 0.05).

There were no significant correlations between the acute post-exercise changes in ΔGH, ΔIGF-1, or Δtestosterone and the changes in Δ1RM and ΔCSA with the different training protocols. However, there was a significant correlation between Δcortisol and ΔCSA in the HL group (*p* = 0.010). Lactate concentration was significantly correlated with Δ1RM and ΔCSA after the training period in the LL group (Table 1). No other significant correlation was found.

**Table 1:** Correlation coefficients between acute hormonal responses (pre to post 15 min), muscle size and strength after 8-week low-load resistance exercise without (LL) and with blood flow restriction (LL-BFR), and high-load resistance exercise (HL).

Groups	Variables	ΔAcute/ΔTraining	r	*p*
**LL**	GH/1RM **(**ng∙ml/kg)	2.1 ± 2.5/17.4 ± 6.3	0.266	0.585
	IGF-1/1RM **(**ng∙ml/kg)	-10427 ± 9600/17.9 ± 5.8	0.153	0.678
	Testosterone/1RM **(**ng∙ml/kg)	-7586 ±15702/17.9 ± 5.8	0.237	0.576
	Cortisol/1RM **(**ng∙ml/kg)	2683 ±3497/17.9 ± 5.8	0.195	0.848
	Lactate/1RM (mmol∙L^-1^/kg)	1.0 ± 0.7/17.9 ± 5.8	0.639	0.044*
	GH/CSA (ng∙ml/mm^2^)	2.1 ±2.5/95.7 ± 291	0.114	0.432
	IGF-1/CSA (ng∙ml/mm^2^)	-10427 ± 9600/84 ± 284	0.311	0.909
	Testosterone/CSA (ng∙ml/mm^2^)	-7586 ± 15702/76.6 ± 277	0.112	0.685
	Cortisol/CSA (ng∙ml/mm^2^)	2683 ± 3497/76.6 ± 277	0.163	0.277

**LL-BFR**	Lactate/CSA (mmol∙L^-1^/mm^2^) GH/1RM **(**ng∙ml/kg)	1.0 ± 0.7/76.6 ± 277 7.0 ± 5.5/34.6 ± 9.2	0.720 0.534	0.017* 0.901
	IGF-1/1RM **(**ng∙ml/kg)	365 ± 263/34 ± 8.8	-0.342	0.817
	Testosterone/1RM **(**ng∙ml/kg)	-5653 ± 13352/34 ± 8.8	0.388	0.060
	Cortisol/1RM **(**ng∙ml/kg)	1956 ± 1950/34 ± 8.8	0.399	0.390
	Lactate/1RM (mmol∙L^-1^/kg)	1.7 ± 0.3/34 ± 8.8	-0.312	0.750
	GH/CSA (ng∙ml/mm^2^)	7.0 ± 5.5/474 ± 220	-0.214	0.980
	IGF-1/CSA (ng∙ml/mm^2^)	365 ± 263/461 ± 212	0.002	0.939
	Testosterone/CSA (ng∙ml/mm^2^)	-5653 ± 13352/461 ± 212	-0.365	0.903
	Cortisol/CSA (ng∙ml/mm^2^)	1956 ± 1950/461 ± 212	-0.008	0.297
	Lactate/CSA (mmol∙L^-1^/mm^2^)	1.7 ± 0.3/461 ± 212	-0.253	0.471

**HL**	GH/1RM **(**ng∙ml/kg)	11.3 ± 10.3/32 ± 8.3	0.020	0.826
	IGF-1/1RM **(**ng∙ml/kg)	35259 ± 37932/32 ± 8.3	0.376	0.932
	Testosterone/1RM **(**ng∙ml/kg)	-1129 ± 2520/31.4 ± 7.3	0.007	0.898
	Cortisol/1RM **(**ng∙ml/kg)	2967 ± 4930/31.4 ± 7.3	0.033	0.587
	Lactate/1RM (mmol∙L^-1^/kg)	1.4 ± 0.6/31.4 ± 7.3	0.644	0.128
	GH/CSA (ng∙ml/mm^2^)	11.3 ± 10.3/541 ± 457	0.140	0.125
	IGF-1/CSA (ng∙ml/mm^2^)	35259 ± 37932/541 ± 457	0.159	0.689
	Testosterone/CSA (ng∙ml/mm^2^)	-1129 ± 2520/513 ± 402	0.236	0.797
	Cortisol/CSA (ng∙ml/mm^2^)	2967 ± 4930/513 ± 402	0.730	0.010*
	Lactate/CSA (mmol∙L^-1^/mm^2^)	1.4 ± 0.6/513 ± 402	0.220	0.763

Mean/±SD - ΔAcute (absolute delta between rest and 15 min after exercise); ΔTraining: absolute delta from pre to post 8 weeks of resistance training for 1RM and the CSA. GH: growth hormone; IGF-1: insulin-like growth factor 1; testosterone; cortisol and lactate concentration. 1RM: knee extension maximal dynamic strength; CSA: quadriceps cross-sectional area. *Significant correlation between acute hormone responses and training effect (p < 0.05).

## Discussion

The main finding of this study was that acute post-exercise changes in systemic anabolic hormone concentrations observed after LL-BFR were not associated with changes in muscle size and strength following an 8-week resistance training program. In addition, our findings demonstrated that adding BFR to a low-load resistance exercise resulted in significantly greater increases in GH concentration (to a similar extent to that observed after conventional high-load resistance exercise). Finally, we observed a similar response pattern between LL-BFR, LL, and HL when considering IGF-1, testosterone, and cortisol concentrations, with no association between training-induced changes in systemic hormones, muscle size and strength adaptations in none of the groups.

For years, the contention was that acute post-exercise (i.e., resistance exercise) responses of hormones such as GH, IGF-1, and testosterone were important drivers of muscle growth. However, even though this assertion is supported by some (Kraemer and Ratamess, 2005; Schoenfeld, 2013), other studies have contrasted it (West et al., 2009). For example, in the West et al.´s (2009) study, in which upper limb muscles were exposed to either a near-basal or a high hormonal concentration, via resistance exercise manipulation (i.e., inclusion of lower-limb exercises for inducing higher hormonal concentrations), the authors observed that hormone concentrations influenced neither myofibrillar protein synthesis nor gains in strength and muscle size. In the same direction, other studies reported no association between exercise-induced elevation in hormone concentrations and the magnitude of gains in strength and muscle size (West et al., 2010; West and Phillips, 2012; Wilkinson et al., 2006).

In the present study, there was an increase in lactate concentration in all groups and an increase in GH concentration 15 min after exercise in the LL-BFR and HL groups. In fact, these findings corroborate some other studies (Kim et al., 2014; Ozaki et al., 2017; Yinghao et al., 2021), however, no association was found between the acute changes in lactate concentrations or GH and training-induced gains in muscle size and strength. Only a significant relationship was observed between changes in lactate concentrations and 1RM or the CSA in the LL group (Table 1). Nonetheless, it has been previously shown that the LL protocol is an insufficient stimulus to induce changes in muscle size or strength when compared with LL-BFR and HL protocols (Laurentino et al., 2012; Takarada et al., 2000).

Testosterone decreased 15 min after exercise in all groups. In addition, there was no association between post-exercise changes in testosterone and the CSA or 1RM, demonstrating that despite a significant reduction in testosterone concentration, muscle size and strength gains were still unaffected, supporting our hypothesis that training-induced gains in muscle mass and strength are not determined by post-exercise hormonal response. Furthermore, similar to our findings, previous studies have investigated the testosterone response to LL-BFR and found no significant changes (Loenneke et al., 2011; Reeves et al., 2006). In this regard, it has been suggested that the non-responsiveness of testosterone to LL-BFR might be related to the low load (e.g., 20% 1RM) used in exercise protocols (Loenneke et al., 2011). On the other hand, divergent from our findings, a recent study (Yinghao et al., 2021) showed LL-BFR-induced increases in testosterone levels with higher pressure (70% of arterial occlusion pressure – AOP) rather than low-pressure (40% of AOP). Those authors speculated that it could have been caused by increased lactate concentrations (Yinghao et al., 2021). Despite that, several studies have shown no association and/or effect on muscular adaptations of a decrease in or maintenance of testosterone response to high-load resistance training (Morton et al., 2016; Zourdos et al., 2016). Together, these findings suggest that physiological exercise-induced changes in testosterone have little or any influence on gains in skeletal muscle size and strength.

There was a positive relationship between acute exercise-induced changes in cortisol and training-induced changes in muscle size in the HL group. This effect has been previously observed and may be related to a stress response associated with high-load resistance exercise (Zourdos et al., 2016). For example, changes in cortisol in response to exercise may reflect the individual effort performed during an intense exercise bout. Although speculative, perhaps individuals who trained hardest, had the greatest change in cortisol, and subsequently had the greatest change in muscle size.

The current data are consistent with the notion that local mechanisms within skeletal muscle are the primary drivers of muscle growth resulting from resistance exercise and are dissociated from post exercise-induced hormonal responses. For example, a decrease in mRNA gene expression of myostatin and an increase in mRNA gene expression of follistatin isoforms, GASP-1 and SMAD-7 via mechanotransduction pathways may play a significant role (Laurentino et al., 2012).

A limitation of this study is that the hormonal concentrations were assessed only at one time point (pre-training). It is possible that these hormones may have changed across time, however, previous studies have demonstrated no change in the time course of systemic hormones (Morton et al., 2016; Wilkinson et al., 2006) and these hormones have been observed to decrease following training (Zourdos et al., 2006). Nevertheless, this study supports the contention that there is no correlation between the post-exercise increase in systemic hormones and muscle growth and strength after resistance training (Morton et al., 2016; West et al., 2010; Wilkinson et al., 2006) and extends this notion to blood-flow-restriction training.

## Conclusions

In conclusion, the present study suggests that despite transient exercise-induced increases in systemic endogenous hormones, these changes did not correlate to the enhanced muscle size and strength observed after LL-BFR. Additionally, hormonal, phenotypical (i.e., CSA), and functional (i.e., 1RM) responses to LL-BFR were comparable to those observed after HL. Finally, all these changes occurred in the absence of significant post-exercise increases in either IGF-1 or testosterone concentrations. These findings reinforce the lack of evidence that post-exercise-induced hormonal changes are important in regulating skeletal muscle hypertrophy and strength gains following blood-flow-restriction and high-load resistance training.
